# Meta-analysis of immunohistochemical prognostic markers in resected pancreatic cancer

**DOI:** 10.1038/bjc.2011.110

**Published:** 2011-03-29

**Authors:** R A Smith, J Tang, C Tudur-Smith, J P Neoptolemos, P Ghaneh

**Affiliations:** 1 Division of Surgery and Oncology, School of Cancer Studies, University of Liverpool, Royal Liverpool University Hospital, 5th Floor Duncan Building, Daulby Street, Liverpool L69 3GA, UK

**Keywords:** immunohistochemistry, molecular, pancreatic cancer, meta-analysis, prognosis

## Abstract

**Background:**

The potential prognostic value of several commonly investigated immunohistochemical markers in resected pancreatic cancer is variably reported. The objective of this study was to conduct a systematic review of literature evaluating p53, p16, smad4, bcl-2, bax, vascular endothelial growth factor (VEGF) and epidermal growth factor receptor (EGFR) expression as prognostic factors in resected pancreatic adenocarcinoma and to conduct a subsequent meta-analysis to quantify the overall prognostic effect.

**Methods:**

Relevant literature was identified using Medline, EMBASE and ISI Web of Science. The primary end point was overall survival assessed on univariate analysis. Only studies analysing resected pancreatic adenocarcinoma were eligible for inclusion and the summary log_e_ hazard ratio (logHR) and variance were pooled using an inverse variance approach. Evidence of heterogeneity was evaluated using the *χ*^2^ test for heterogeneity and its impact on the meta-analysis was assessed by the *I*^2^ statisic. Hazard ratios greater than one reflect adverse survival associated with positive immunostaining.

**Results:**

Vascular endothelial growth factor emerged as the most potentially informative prognostic marker (11 eligible studies, *n*=767, HR=1.51 (95% confidence interval, CI=1.18–1.92)) with no evidence of any significant publication bias (Egger's test, *P*=0.269). Bcl-2 (5 eligible studies, *n*=314, HR=0.51 (95% CI=0.38–0.68)), bax (5 studies, *n*=274, HR=0.63 (95% CI=0.48–0.83)) and p16 (3 studies, *n*=229, HR=0.63 (95% CI=0.43–0.92)) also returned significant overall survival differences, but in smaller patient series due to a lack of evaluable literature. Neither p53 (17 studies, *n*=925, HR=1.22 (95% CI=0.96–1.56)), smad4 (5 studies, *n*=540, HR=0.88 (95% CI=0.61–1.27)) nor EGFR (4 studies, *n*=250, HR=1.35 (95% CI=0.80–2.27)) was found to represent significant prognostic factors when analysing the pooled patient data. There was evidence of significant heterogeneity in four of the seven study groups.

**Conclusion:**

These results support the case for immunohistochemical expression of VEGF representing a significant and reproducible marker of adverse prognosis in resected pancreatic cancer.

Pancreatic ductal adenocarcinoma is characterised by its singularly aggressive tumour biology and unfavourable patient outcomes. Despite overall 5-year survival rates of <5%, previous randomised trials have demonstrated that for patients presenting with localised disease, resection with administration of adjuvant chemotherapy is associated with 5-year survival rates of over 20% (Neoptolemos *et al*, 2004; Oettle *et al*, 2007).

Reliable identification of molecular prognostic markers is important in order to facilitate the rational selection of potential therapeutic targets in the development of novel cancer therapies and to allow meaningful and reproducible risk stratification as part of clinical trials. There is marked disparity in the literature between individual studies as to the relative prognostic impact of several immunohistochemical tissue markers in pancreatic cancer. This may, in part, be explained by heterogeneity in patient selection due to inclusion of resected and unresected patients in survival analyses or inclusion of mixed tumour types and laboratory methodology when comparing different studies. The objective of the present study was to conduct a systematic review and meta-analysis of published literature investigating the commonly reported immunohistochemical prognostic markers in resected primary tumour material from patients with pancreatic adenocarcinoma and to identify potential sources of heterogeneity when comparing the results of individual studies.

## Materials and methods

### Search strategy

Medline, EMBASE and ISI Web of Science were searched to identify potentially relevant published literature. No chronological search criteria were applied. Existing systematic reviews and reference lists were also checked for any potentially relevant additional studies. The most widely investigated and biologically relevant immunohistochemical tissue markers for pancreatic cancer were selected for meta-analysis. These comprised p53, smad4, p16, bcl-2, bax, vascular endothelial growth factor (VEGF) and epidermal growth factor receptor (EGFR).

### Selection criteria

The following criteria were used to search English language articles and abstracts: ‘(*marker*)’ AND (‘pancreas’ OR ‘pancreatic’) AND (‘survival’ OR ‘prognosis’ OR ‘prognostic’). Each search was repeated for individual markers by substituting the name of marker of interest along with relevant synonyms: ‘p53’ OR ‘TP53’ ‘p16’ OR ‘p16^*^’ OR ‘CDKN2A’ ‘smad4’ OR ‘smad-4’ OR ‘smad^*^’ ‘DPC4’ OR ‘DPC-4’ OR ‘DPC^*^’ ‘bcl-2’ OR ‘bcl2’ OR ‘bcl’ OR ‘bcl^*^’ ‘bax’ ‘vascular endothelial growth factor’ OR ‘VEGF’ OR ‘VEGF^*^’ ‘epidermal growth factor receptor’ OR ‘EGFR’ OR ‘c-erbB^*^’ OR ‘erbB^*^’ OR ‘HER^*^’. The search was performed in November 2009. Abstracts were initially checked for relevance and the full article was retrieved for all potentially eligible studies. Where part or all of the same patient series was included in more than one publication, only the more recent or most complete study was included in the analysis in order to avoid duplication of the same survival data.

The following inclusion criteria were used to select literature: only cases of resected pancreatic adenocarcinoma analysed, immunohistochemical expression assessed in resected primary tumour material, dichotomised univariate survival analysis reported (i.e. positive *vs* negative staining) and overall survival times used in analysis. For the analysis of VEGF, only studies investigating the prognostic value of VEGF-A expression were included. Authors were contacted for unpublished results in cases where insufficient survival data were reported to estimate the log_e_ hazard ratio (logHR) and variance. Due to the minority of studies reporting multivariable analyses, no attempt was made to use any adjusted survival data as part of this meta-analysis (i.e. only univariate survival data were extracted).

### End points

The primary outcome measure was overall survival (i.e. date of resection to date of death). Additional details were also collected in order to identify potential sources of heterogeneity. These included the specific primary antibody (and dilution) used for immunohistochemistry, the scoring criteria used to define positive staining and relevant clinico-pathological data. An assessment of study methodology was made according to previously defined criteria (Hayden *et al*, 2006; McShane *et al*, 2006). These principles were used to define 20 individual study characteristics, which were deemed to be key factors to report in an immunohistochemical prognostic study (Table 1). For any criterion not fulfilled according to the information outlined in the article, one point was deducted from a maximum of 20 and the final score was recorded as a percentage. The eligibility criteria and quality scoring were assessed by two independent investigators. Any disagreement was resolved by discussion.

### Statistical analysis

Previously reported indirect methods were utilised for extracting the logHR and variance due to the paucity of prognostic literature, which report these values directly (Parmar *et al*, 1998; Williamson *et al*, 2002; Tierney *et al*, 2007). These values were either calculated from the HR and 95% confidence interval (CI) where quoted, the log rank *P*-value, or from the Kaplan–Meier survival curves directly. The software used for these indirect calculations was designed by Matthew Sydes and Jayne Tierney of the Medical Research Council Clinical Trials Unit, London, UK (Tierney *et al*, 2007). The logHR and variance for individual studies were entered into RevMan 4.2 (Cochrane collaboration, Oxford, UK) and pooled using a random effects inverse variance approach. The overall prognostic effect of positive immunostaining was recorded as an HR and 95% CI (i.e. an HR>1 reflecting adverse survival associated with positive immunostaining). Heterogeneity was assessed using a *χ*^2^ test for heterogeneity with a *P*-value of <0.10 taken to reflect the presence of significant heterogeneity. The *I*^2^ statistic was calculated to quantify the degree of heterogeneity (Higgins and Thompson, 2002). A *P*-value of <0.050 was taken to reflect significance for all other analyses. Publication bias was assessed by inspection of the funnel plot with Egger's regression. Continuous data were compared using Spearman's rank correlation and two-sided Mann–Whitney testing for categorical data.

## Results

### VEGF

The initial search returned a total of 255 studies. Following review of these abstracts, 20 potentially relevant studies were identified as eligible of which nine were excluded for the following reasons: duplicated series of patients (Ikeda *et al*, 1999; Niedergethmann *et al*, 2000; Tang *et al*, 2001), only VEGF-C and/or VEGF-D analysed (Kurahara *et al*, 2004; Zhang *et al*, 2007), no dichotomised univariate survival analysis reported (Ellis *et al*, 1998; Fujioka *et al*, 2001), mix of resected and unresected cases included in survival analysis (Chung *et al*, 2006) and only VEGF receptor status analysed (Büchler *et al*, 2002).

**Figure 1:**
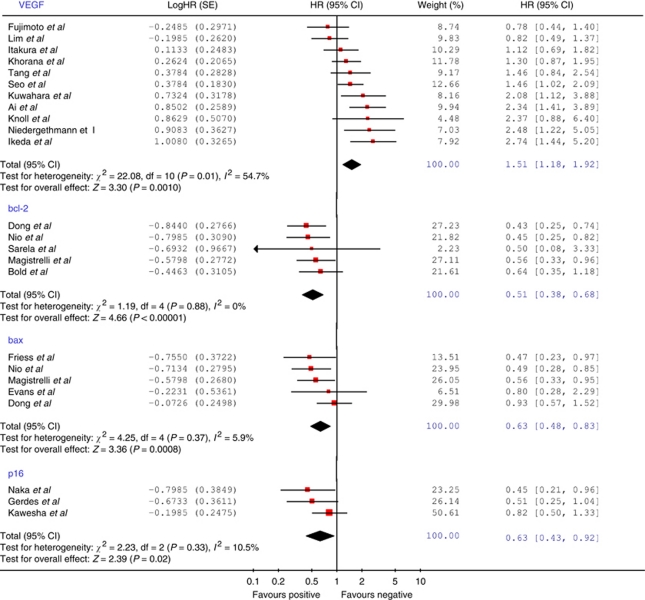
Forrest plot to assess overall effect of VEGF, bcl-2, bax and p16 expression on survival.

**Figure 2:**
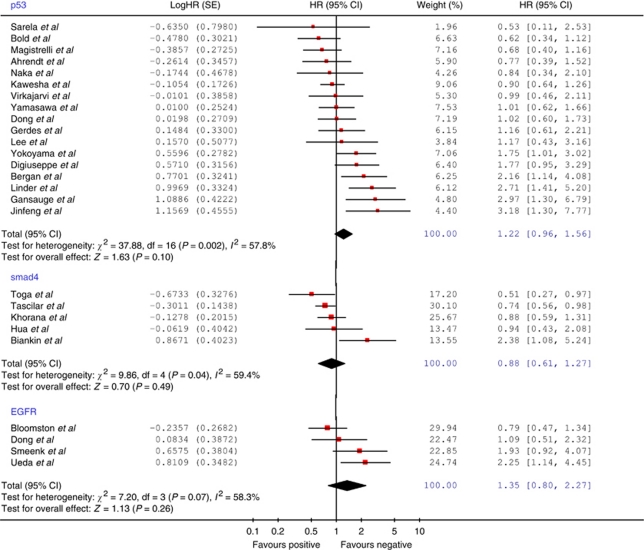
Forrest plot to assess overall effect of p53, smad4 and EGFR expression on survival.

The 11 eligible studies (all retrospective) included a total of 767 patients with a median number of 62 patients per study (range=19–142). Table 2 outlines the demographic, clinico-pathological, methodological and outcome characteristics of these studies. The median quality score was recorded as 70% (range=60–95%). There was no significant difference in median quality scores between significant and non-significant studies (Mann–Whitney, *P*=0.516). Similarly, there was no significant correlation between study size and quality scores (Spearman's *ρ*=0.139, *P*=0.698). Figure 1 illustrates the Forrest plot for the survival data. Significant heterogeneity was demonstrated according to Cochran's *χ*^2^ test (*χ*^2^=22.08, *P*=0.01; *I*^2^=54.7%). The combined HR was recorded as 1.51 (95% CI=1.18–1.92), indicating that positive immunostaining for VEGF was significantly associated with adverse survival in the pooled patient group. When assessing the funnel plot for this analysis (Figure 2), the data points approximated a symmetrical distribution (Egger's test, *P*=0.269), indicating that publication bias is unlikely to be a significant confounding factor in describing this relationship.

The median proportion of patients classified as VEGF positive in the included studies was recorded as 60% (range=32–71%). The proportion of VEGF positive cases reported in each study failed to exhibit any correlation with the assessment of methodological quality (Spearman's *P*=0.491) or the % cutoff used to define positive immunostaining (Spearman's *P*=0.388). Only six studies reported the proportion of patients who received any form of adjuvant therapy (Table 2) and administered treatment modalities included a mix of both chemotherapy and chemoradiation. No studies reported use of any neoadjuvant therapy and only a single study reported use of intra-operative radiotherapy (Ikeda *et al*, 2001). Of the five studies that reported positive VEGF expression as a significant adverse prognostic variable, only three conducted some form of multivariate analysis. These three analyses included a variety of disparate covariates alongside VEGF. However, each reported that VEGF expression retained statistical significance.

### bcl-2

The initial search returned a total of 232 abstracts of which 16 potentially eligible articles were retrieved. A total of 11 were excluded for the following reasons: duplicated series of patients (Nio *et al*, 2001a), mix of resected and unresected cases included (Gansauge *et al*, 1998; Mäkinen *et al*, 1998; Ohshio *et al*, 1998; Hu *et al*, 1999), inclusion of ampullary tumours (Sinicrope *et al*, 1996), no dichotomised univariate survival analysis conducted (Evans *et al*, 2001; Stipa *et al*, 2002; Sun *et al*, 2002) and insufficient survival data reported for indirect estimation of logHR and variance (Friess *et al*, 1998; Campani *et al*, 2001).

**Figure 3:**
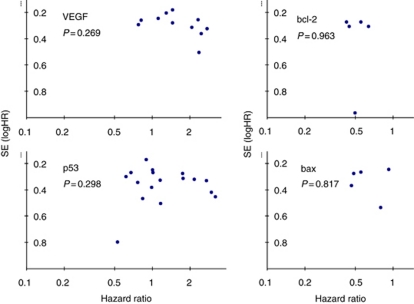
Funnel plots to assess publication bias for VEGF, bcl-2, bax and p53 meta-analyses. *Note*: *P*-values for result of Egger's regression to assess publication bias.

The five eligible studies included a total of 314 patients with a median number of 63 patients per study (range=52–70) (Table 2). The median quality score was recorded as 75% (range=65–85%) and the median proportion of bcl-2 positive cases was 33% (range=12–67%). Figure 2 illustrates the Forrest plot for the pooled survival data. There was no evidence of any significant heterogeneity (*χ*^2^=1.19, *P*=0.88). The combined HR was recorded as 0.51 (95% CI=0.38–0.68), indicating a significant association between positive bcl-2 immunostaining and more favourable survival in the pooled patient group. Despite the limited number of studies included, the funnel plot for this analysis failed to demonstrate any obvious asymmetry (Figure 3). Three studies reported use of either adjuvant chemotherapy or chemoradiation and a single study (Bold *et al*, 1999) also reported use of neoadjuvant chemoradiation in 43 out of the 70 patients analysed. Of the two studies rejected due to incomplete survival data (Friess *et al*, 1998; Campani *et al*, 2001), both failed to observe any significant prognostic effect associated with bcl-2 expression. Neither study reported the direction of the prognostic effect.

### bax

The initial search yielded 76 studies. Following review of the abstracts, a total of seven potentially eligible articles were identified. Two of these were excluded due to either a duplicated patient series (Hashimoto *et al*, 2005) or the inclusion of periampullary cancers of non-pancreatic origin in the survival analysis (Tomazic *et al*, 2004). Three of the five eligible studies investigated the prognostic effect of both bcl-2 and bax and were, therefore, included in both meta-analyses (Magistrelli *et al*, 2006; Nio *et al*, 2001b; Dong *et al*, 2005b).

The five eligible studies investigating bax included a total of 274 patients with a median number of 60 patients per study (range=23–67) (Table 2). The median quality score was 65% (range=55–85%) and the median proportion of bax positive cases was 54% (range=26–83%). Figure 2 illustrates the Forrest plot for the pooled survival data. There was no evidence of any significant heterogeneity (*χ*^2^=4.25, *P*=0.37; *I*^2^=5.9%). The combined HR was recorded as 0.63 (95% CI=0.48–0.83) and the funnel plot for this analysis is shown in Figure 3.

### p16

The initial search returned 91 studies, seven of which were potentially relevant. Following review of these seven articles, three fulfilled all of the eligibility criteria. The remaining studies were rejected due to the inclusion of unresected cases (Hu *et al*, 1997; Biankin *et al*, 2002), no IHC used in tissue analysis (Ohtsubo *et al*, 2003) or only disease-free survival times reported (Jeong *et al*, 2005). A total of 229 patients were included in the pooled analysis. There was no evidence of any significant heterogeneity across the three included studies (*χ*^2^=2.23, *P*=0.33; *I*^2^=10.5%). A combined HR of 0.63 (95% CI=0.43–0.92) was obtained, indicating a significant association between p16 expression and more favourable survival.

### p53

The initial search returned a total of 337 studies. Following review of these abstracts, 58 potentially relevant studies were retrieved of which 17 fulfilled all of the inclusion criteria. The remaining studies were rejected for the following reasons: duplicated series of patients (Dergham *et al*, 1997a; Dong *et al*, 1998b; Nio *et al*, 1998; Nio *et al*, 1999; Dong *et al*, 2000; Linder *et al*, 2001; Nio *et al*, 2001a), no dichotomised univariate survival analysis conducted (Sessa *et al*, 1998; Karademir *et al*, 2000; Evans *et al*, 2001; Fujioka *et al*, 2001; Gazzaniga *et al*, 2001; Biankin *et al*, 2002; Dang *et al*, 2002; Hashimoto *et al*, 2005; Dong *et al*, 2007; Smeenk *et al*, 2007), no IHC used in tissue analysis (Weyrer *et al*, 1996; Li *et al*, 1999; Yamaguchi *et al*, 2000; Ohshio *et al*, 2002; Dong *et al*, 2003), unresected cases included in survival analysis (Zhang *et al*, 1994; Aizawa *et al*, 1996; Lundin *et al*, 1996; Coppola *et al*, 1998; Dergham *et al*, 1998; Mäkinen *et al*, 1998; Ohshio *et al*, 1998; Hu *et al*, 1999; Takikita *et al*, 2009), mix of different tumour types included (Sinicrope *et al*, 1996; Sato *et al*, 1997; Gansauge *et al*, 1998; Yu *et al*, 2004), only disease-free survival reported (Jeong *et al*, 2005) and insufficient survival data reported (Dergham *et al*, 1997b; Campani *et al*, 1999; Stipa *et al*, 2002; Hermanova *et al*, 2009).

The 17 eligible studies included a total of 925 patients with a median number of 48 patients per study (range=26–157) (Table 3). Nuclear staining of p53 was used for scoring in all cases. Five studies (29%) reported a significant adverse association between p53 expression and survival. The median quality score was recorded as 65% (range=45–90%) and the median proportion of patients exhibiting positive p53 immunostaining was 47% (range=25–68%). There was no significant association between the IHC cutoff score used and the proportion of cases classified as p53 positive (Spearman's *ñ*=0.389, *P*=0.206). Furthermore, there was no significant difference in median quality scores between significant and non-significant studies (Mann–Whitney, *P*=0.243).

Figure 2 illustrates the Forrest plot for the survival data. There was no evidence of any significant publication bias (Egger's test, *P*=0.298). Significant heterogeneity was demonstrated according to Cochran's *χ*^2^ test (*χ*^2^=37.88, *P*=0.002; *I*^2^=57.8%). The combined HR was recorded as 1.22 (95% CI=0.96–1.56), indicating no significant overall association between p53 expression and survival. Of the four studies excluded due to incomplete reporting of survival data, only one reported a significant association between p53 expression and survival (Stipa *et al*, 2002).

### smad4

The initial search returned 81 studies. Following review of these abstracts, five potentially relevant studies were identified, which were all found to be eligible for analysis. The combined number of patients was 540 with a median of 88 patients per study (range=34–249) (Table 3). The median quality score was 75% (range=60–95%) and the median proportion of patients exhibiting positive smad4 immunostaining was 45% (range=15–76%). Figure 2 illustrates the Forrest plot. There was evidence of significant heterogeneity across the included studies (*χ*^2^=9.86, *P*=0.04; *I*^2^=59.4%). A combined HR of 0.88 (95% CI=0.61–1.27) was recorded, indicating no significant overall association between smad4 expression and survival in the pooled patient group.

### EGFR

The initial search identified 324 studies. Following review of these abstracts, 10 potentially relevant articles were retrieved. Six of these studies were rejected for the following reasons: duplicated series of patients (Uegaki *et al*, 1997; Ueda *et al*, 2006), no dichotomised univariate survival analysis conducted (Yamanaka *et al*, 1993; Zhang and Yuan, 2002) and unresected cases included in analysis (Gansauge *et al*, 1998; Takikita *et al*, 2009). The four eligible studies included a total of 250 patients (Table 3). Only a single study reported a significant relationship between EGFR expression and survival (Ueda *et al*, 2004). The median quality score was 70% (range=65–70%). Figure 2 illustrates the Forrest plot for the pooled data. Significant heterogeneity was demonstrated (*χ*^2^=7.20, *P*=0.07). The combined HR was recorded as 1.35 (95% CI=0.80–2.27), indicating no significant overall association between EGFR expression and survival.

## Discussion

Previous meta-analyses of studies investigating the prognostic value of molecular markers have been published for different malignancies. These include VEGF (Delmotte *et al*, 2002; Kyzas *et al*, 2005a; Des Guetz *et al*, 2006), bcl-2 (Martin *et al*, 2003; Callagy *et al*, 2008) and p53 (Kyzas *et al*, 2005b; Malats *et al*, 2005). To date, no such meta-analysis has been undertaken for any studies evaluating immunohistochemical prognostic markers in resected pancreatic cancer.

Meta-analysis of prognostic literature is associated with a number of inherent limitations. One of these key limitations is the general prevalence of retrospective study design in this setting. None of the studies included in the current meta-analysis specified a prospective design and archived paraffin-embedded tumour material was utilised for IHC in all cases. This indicates that availability of tissue is invariably the main determinant of study size rather than any specific considerations relating to adequate statistical power in order to reliably detect a prognostic effect for the marker of interest. The availability and adequacy of corresponding clinico-pathological data is also a significant consideration in retrospective studies of this type and we identified several studies reporting incomplete datasets with regard to histopathological details. Alongside this, an additional hindrance to meta-analysis of prognostic literature is the general lack of multivariable survival data. This is usually attributable to the fact that the number of patients included in each study is typically small, precluding any meaningful attempt at analysing multiple covariates.

Additional challenges in the interpretation and comparison of immunohistochemical prognostic studies include variability in patient selection (i.e. resected and unresected cases, inclusion of non-pancreatic periampullary tumours), disparate immunohistochemical criteria used for prognostic classification, bias associated with the statistical approach to analysis of survival data (e.g. selection of data-driven cutoff values for continuous variables), incomplete reporting of survival data, duplicated patient series and publication bias arising as a result of selective reporting of ‘positive’ studies (Altman, 2001). In order to overcome some of these comparative difficulties, specific inclusion criteria were applied in order to select studies for meta-analysis. Only studies including resected pancreatic adenocarcinoma were included in order to avoid any confounding effects on survival associated with differing proportions of resected and unresected cases. Any studies including periampullary tumours of non-pancreatic origin were also excluded due to the disparity in survival outcomes characteristically associated with ampullary, duodenal and bile duct adenocarcinomas when compared with pancreatic adenocarcinoma (Riall *et al*, 2006). Furthermore, in cases where part or all of the same patient series was included in more than one publication, only the more recent or most complete study was included in the analysis in order to avoid duplicating the same patient data for the immunohistochemical marker of interest. For those studies where insufficient survival data was reported to generate indirect calculations for the logHR and variance, authors were contacted for additional survival data. However, in all cases the authors were either unable to provide any supplementary data or no response was received. The only supplementary raw data obtained was for two studies previously conducted at our own institution (Kawesha *et al*, 2000; Evans *et al*, 2001). Therefore, no subsequent attempt to request individual patient survival data for all eligible studies was undertaken, although this would have been potentially beneficial.

When analysing the overall relationships between individual study size, reported prognostic significance and methodological quality scores in the present study, there was a significant trend towards superior methodological quality in larger studies as one might reasonably expect, despite the fact that study size itself was not one of the criteria used for quality scoring. When considering the overall effect of potential publication bias in this analysis, only a minority of studies (21 out of 50) actually reported a statistically significant prognostic result. Furthermore, the funnel plots and Egger's tests for the individual analyses, although more difficult to interpret when fewer studies were included, were not generally indicative of any strong publication bias.

Vascular endothelial growth factor emerged as the most potentially informative immunohistochemical prognostic marker from the pooled data. Vascular endothelial growth factor comprises four ligands (VEGF-A, VEGF-B, VEGF-C and VEGF-D), which exhibit specific binding profiles with three transmembrane VEGF receptors (VEGF-I, -II and -III) and promote intracellular tyrosine kinase cascades when activated. The VEGF-A (usually referred to simply as VEGF) mediates the key pro-angiogenic properties of proliferation and migration of endothelial cells along with increasing vascular permeability (Yamazaki and Morita, 2006; Dallas *et al*, 2007). Alternate gene splicing results in a number of VEGF-A isoforms of differing amino-acid lengths, the smaller of which (e.g. 121 and 165) are secreted while the larger (e.g. 189 and 206) remains cell associated. VEGF-C and VEGF-D are implicated in the process of lymphangiogenesis (Achen and Stacker, 2008) while the function of VEGF-B is incompletely understood (Nash *et al*, 2006). Pancreatic cancer cells have been demonstrated to express both VEGF ligand and its receptors, implicating a potential VEGF-mediated autocrine loop in the proliferation of pancreatic malignancy (Büchler *et al*, 2002).

The results from the present study demonstrate that, despite variability between eligible studies as to the relative prognostic impact of VEGF expression in resected pancreatic adenocarcinoma, the observed survival trend is concordant with that reported for other malignancies in similar meta-analyses (Delmotte *et al*, 2002; Kyzas *et al*, 2005a, 2005b; Des Guetz *et al*, 2006). When comparing the value for the pooled HR identified in the present study (1.51 (95% CI=1.18–1.92)) with the above referenced studies, the order of magnitude for this effect is also broadly comparable for that quoted for both lung cancer (1.48 (95% CI=1.27–1.72)) and colorectal cancer (1.65 (95% CI=1.27–2.14)).

Significant heterogeneity was observed when analysing the logHR estimates from the eligible studies. Evaluation of the relevant methodological and clinico-pathological characteristics of each study revealed a number of potential sources of heterogeneity in study methodology. Nine studies reported use of commercially available anti-VEGF primary antibodies, all of which exhibit broadly comparable binding characteristics with the common splice variants of VEGF-A. When analysing the concentrations of primary antibody utilised, most studies reported comparable dilution ratios. However, the concentration was not specified in two studies. This issue is potentially relevant for the study reporting use of the lowest primary antibody dilution (Lim *et al*, 2004) as this was one of only two studies, which indicated a contradictory prognostic effect when compared with the overall group (i.e. a non-significant trend towards adverse survival with negative VEGF immunostaining).

When reviewing the immunohistochemical criteria used for VEGF scoring, the majority of studies reported a scoring system based on cytoplasmic staining of tumour cells. Where the distribution of immunostaining used for scoring was not explicitly stated in the text (i.e. cytoplasmic, membranous, nuclear, stromal, etc.), the figures of representative VEGF staining presented in the relevant studies were all strongly indicative of cytoplasmic staining being used to define positive VEGF expression in cancer cells. All studies with one exception utilised a system of dichotomising patients according to the percentage of positively stained cells present. Despite the range of values used to define VEGF positivity across the included studies, there was no evidence of any significant association between the % cutoff value used and the corresponding proportion of VEGF positive patients reported. Furthermore, if including only the six studies, which used a standardised cutoff value of >10% for meta-analysis, the significance of the association between VEGF staining and adverse survival was unchanged (HR=1.62 (95% CI=1.09–2.40)−random effects). These observations indicate that differences in the specific scoring criteria used for immunohistochemical classification appear unlikely to have a significant confounding effect in describing the underlying relationship between VEGF expression and survival observed for the overall group.

Broadly comparable demographic and histological tumour characteristics were observed across the eligible VEGF studies, indicating that similar patient populations were evaluated in the combined analysis. Data relating to adjuvant therapy was only reported in 6 out of 11 studies and the treatment modalities included a mix of both chemotherapy and chemoradiation. Importantly, no studies reported any policy of selection of patients for adjuvant therapy based on VEGF tumour expression as immunohistochemical evaluation was undertaken on a retrospective basis in all cases. This was equally true for studies evaluating the other markers of interest.

Both bcl-2 and bax emerged as potentially relevant immunohistochemical prognostic factors. These proteins belong to the bcl-2 family and regulate apoptosis by mediating cytosolic release of cytochrome C from mitochondria in response to cellular stress. Cytochrome C binds to APAF-1 and cleaves caspase-9 into its active form, thereby initiating the activation of executioner caspases resulting in cytoskeletal degradation and cell death (Hamacher *et al*, 2008). The bcl-2-associated X protein (bax) promotes release of cytochrome C and consequently exhibits pro-apoptotic properties. In contrast, bcl-2 inhibits mitochondrial release of cytochrome C and has anti-apoptotic effects as a result. The finding that bax expression is associated with more favourable survival in resected pancreatic cancer is, therefore, concordant with its physiological role. The observation that the same relationship is consistently seen for bcl-2 expression appears paradoxical. However, this finding is mirrored in other malignancies (Martin *et al*, 2003; Callagy *et al*, 2008) and it is believed that a complex interaction of competitive dimerisations between pro- and anti-apoptotic proteins governs the cell's fate in response to apoptotic stimuli (Westphal and Kalthoff, 2003). It is difficult to draw any reliable conclusions from the current meta-analysis of bcl-2 and bax for the pancreatic literature due to the limited number of evaluable studies. However, the overall trend towards both bax and bcl-2 expression being associated with more favourable survival outcomes is generally consistent with the findings seen in other malignancies.

The tumour suppressor gene p16 (CDKN2A) has a key role in pancreatic carcinogenesis (Schutte *et al*, 1997). p16 is a cell-cycle checkpoint protein, which binds to cyclin-dependent kinases resulting in cell-cycle arrest at the G1/S checkpoint. The observation that positive immunostaining for p16 appears to represent a favourable prognostic feature is, therefore, also consistent with its tumour suppressor function. However, the small number of eligible studies included in this analysis again precludes any meaningful conclusions regarding the reproducibility of p16 expression as a reliable marker of prognosis in resected pancreatic cancer.

Of the various factors evaluated in the present study, the tumour suppressor protein p53 was found to represent the most extensively investigated immunohistochemical prognostic marker. It also exhibited a significant degree of heterogeneity in the reported association between immunostaining and survival for individual studies. Although the overall trend was towards overexpression of p53 resulting in adverse survival for the pooled data, this did not reach significance and there is no obvious explanation for the contradictory results seen between the various studies. The majority of studies used either the monoclonal DO-7, DO-1 or polyclonal CM-1 primary antibodies, which all exhibit immunoreactivity with both wild-type and mutant forms of p53. Due to the increased stability of mutant p53, most of the nuclear immunostaining seen reflects the presence of the mutant rather than wild-type p53 protein. Despite the marked differences between studies in terms of the proportion of cases classified as p53 positive, reported primary antibody dilutions used and cutoff values selected for immunohistochemical scoring, there was no clear association between any of these factors and either the direction of the prognostic effect or the reported magnitude of the HR, which might potentially explain the disparity in survival trends. As a result of these findings, immunohistochemical overexpression of p53 cannot be recommended as a reliable or reproducible marker of prognosis in resected pancreatic cancer from the available evidence.

The smad4 (or DPC4) protein is a central component of the intracellular signalling pathway for transforming growth factor *β* (TGF-*β*), and loss of smad4 expression represents an important event in the progression of PanINs to invasive malignancy (Wilentz *et al*, 2000). The results from the analysis of the five studies evaluating smad4 expression again demonstrate unexplained heterogeneity in the reporting of the prognostic effect of this marker. Biankin *et al* reported an entirely contradictory survival trend to the other four studies with loss of smad4 expression being associated with significantly *improved* patient survival despite use of the same primary antibody and otherwise broadly comparable study methodology and patient groups. This survival trend appears at odds with the accepted tumour suppressor role of smad4 in mediating the inhibitory signalling associated with the TGF-*β* pathway. Despite the fact that the patient series reported by Biankin *et al* only accounts for 8% of all patients included in the combined analysis and 14% of the weighting allocated to the pooled survival data, the discrepancy in the results is such that sufficient heterogeneity is introduced to require a random effects approach resulting in a non-significant result for the overall analysis. These findings further underline the difficulties in making any reliable conclusions regarding the relative prognostic value of immunohistochemical markers when analysed in limited patient series.

Epidermal growth factor receptor is the cell surface receptor for a family of extracellular ligands, which include EGF and TGF-*α* and is coded for by the c-erbB1 proto-oncogene. Activation of EGFR stimulates intracellular tyrosine kinase phosphorylation with consequent activation of a number of signalling cascades including the MAPK (mitogen-activated protein kinase) and Akt (protein kinase) pathways, which promote cell proliferation (Ciardello and Tortora, 2008). The analysis of the four eligible studies included in the current meta-analysis again fails to make a strong case for tumoural overexpression of EGFR representing a reproducible prognostic marker. However, the laboratory methodologies reported in the four studies demonstrated more marked variability (e.g., use of four different EGFR primary antibodies) when compared with some of the other analyses.

Despite the inherent limitations of meta-analysing prognostic literature, the findings from the present study suggest that VEGF represents the most consistently reproducible molecular marker with prognostic value in resected pancreatic adenocarcinoma. This result is concordant with existing meta-analyses, which implicate a similar prognostic role for VEGF expression in other malignancies and lend further weight to the assertion that angiogenesis is a key determinant in driving pancreatic cancer progression. For several of the other markers evaluated in this study, directly contradictory prognostic effects were commonly observed with significant variability in the proportions of positive immunostaining reported, despite often broadly comparable patient groups and study methodologies. These results provide further evidence to suggest that in order to make reliable conclusions regarding immunohistochemical prognostic factors and to identify the relevance with which these factors can be translated into clinical use (e.g., individualised patient selection for adjuvant therapy modalities), large collaborative studies collecting tissue as part of prospective multicentre trials, with standardised approaches to both laboratory and statistical methodology, represent the optimal strategy to achieve these goals in the future (e.g. Farrell *et al*, 2009; Manuyakorn *et al*, 2010).

## Figures and Tables

**Table 1:** Methodological scoring criteria used

*Study group*	
Study population adequately described	
Gender/age	1 Point
Histology	1 Point
Period of recruitment	1 Point
Inclusion/exclusion criteria used	1 Point
	
*Study attrition*	
>90% of cases identified included in final analysis	1 Point
Reasons for attrition/loss to follow-up given	1 Point
Peri-operative mortality details	1 Point
	
*Scientific methodology*	
IHC methodology outlined	
Details of 1°/2° Abs used	1 Point
Concentration of 1° Abs used	1 Point
Positive/negative controls outlined	1 Point
Description of scoring technique	
>1 independent scorer	1 Point
Scorers blinded to clinical data	1 Point
Criteria for positivity clearly outlined	
Distribution (cytoplasm *vs* membranous *vs* nuclear)	1 Point
% positive cells for immunostaining classification	1 Point
	
*Confounding factors considered*	
Adjuvant therapy details provided	1 Point
Histological breakdown according to IHC staining	1 Point
	
*Statistical analysis*	
HR (confidence interval) provided	1 Point
Exact *P*-value quoted	1 Point
Numbers at risk for Kaplan–Meier curves	1 Point
Number of censored cases recorded	1 Point

Abbreviations: HR=hazard ratio; IHC=immunohistochemical.

**Table 2:** Methodological and clinico-pathological data for eligible prognostic studies evaluating VEGF, bcl-2, bax and p16

**Reference**	***n***	**HR (95% CI)**	**Significant**	**1° Ab (+dilution)**	**IHC +ve**	**IHC cutoff (%)**	**Male**	**Age**	**N1**	**T3/T4**	**Well**	**Mod.**	**Poor**	**Adjuvant therapy**
*VEGF*														
Itakura *et al* (1997)	75	1.12 (0.69–1.82)	No	NC (30 *μ*g ml^–1^)	48 (64)	>10	46 (61)	62	47 (63)	43 (57)	13 (17)	44 (59)	18 (24)	NS
Fujimoto *et al* (1998)	50	0.78 (0.44–1.40)	No	Santa Cruz A20 (1 : 200)	28 (40)	NS	28 (56)	62	29 (58)	34 (68)	9 (18)	31 (62)	10 (20)	NS
Seo *et al* (2000)	142	1.46 (1.02–2.09)	Yes	Santa Cruz (NS)	94 (66)	>30	79 (56)	64	95 (67)	NS	NS	NS	NS	NS
Ikeda *et al* (2001)	48	2.74 (1.44–5.20)	Yes	Santa Cruz (1 : 200)	31 (65)	>10	37 (77)	64	24 (50)	40 (83)	15 (31)	28 (58)	5 (11)	48 (100)
Knoll *et al* (2001)	19	2.37 (0.88–6.40)	No	R&D Ab293NA (1 : 200)	13 (68)	>5	11 (58)	58	18 (95)	1 (5)	1 (5)	12 (63)	6 (32)	0 (0)
Niedergethmann *et al* (2002)	70	2.48 (1.22–5.05)	Yes	Santa Cruz (1 : 200)	28 (40)	>10	42 (60)	63	41 (59)	NS	25 (36)	45 (64)	22 (31)	
Kuwahara *et al* (2003)	55	2.08 (1.12–3.88)	Yes	Santa Cruz sc152 (1 : 200)	39 (71)	>50	34 (62)	64	30 (55)	40 (73)	13 (24)	33 (60)	9 (16)	NS
Lim *et al* (2004)	72	0.82 (0.49–1.37)	No	Santa Cruz (1 : 2000)	23 (32)	>10	43 (60)	60	38 (53)	59 (82)	14 (19)	44 (61)	14 (19)	26 (36)
Khorana *et al* (2005)	124	1.30 (0.87–1.95)	No	Zymed (1 : 50)	70 (56)	>5	69 (56)	67	56 (45)	69 (58)	23 (19)	52 (43)	45 (38)	88 (79)
Tang *et al* (2006)	50	1.46 (0.84–2.54)	No	NS (2 *μ*g ml^–1^)	25 (50)	>10	25 (50)	63	39 (78)	25 (50)	15 (30)	31 (62)	4 (8)	NS
Ai *et al* (2008)	62	2.34 (1.41–3.89)	Yes	Neomarkers (NS)	37 (60)	>10	36 (58)	65	49 (79)	32 (52)	17 (27)	15 (24)	30 (48)	0 (0)
														
*bcl-2*														
Bold *et al* (1999)	70	0.64 (0.35–1.18)	No	DAKO (NS)	23 (33)	>25%	36 (51)	64	32 (46)	NS	15 (22)	37 (55)	15 (22)	19 (27)
Nio *et al* (2001b)	66	0.45 (0.25–0.82)	Yes	DAKO M0887 (1 : 100)	16 (24)	>5%	31 (47)	66	54 (82)	NS	33 (50)	29 (44)	4 (6)	36 (55)
Magistrelli *et al* (2006)	67	0.56 (0.33–0.96)	Yes	DAKO c124 (1 : 40)	45 (67)	>5%	45 (67)	63	34 (51)	40 (62)	14 (21)	28 (42)	15 (22)	30 (45)
Sarela *et al* (2002)	52	0.50 (0.08–3.33)	No	DAKO (1 : 40)	6 (12)	>10%	27 (52)	64	40 (78)	49 (94)	11 (22)	24 (47)	16 (31)	NS
Dong *et al* (2005b)	59	0.43 (0.25–0.74)	Yes	DAKO M124(1 : 100)	21 (36)	>5%	19 (32)	55	54 (82)	NS	19 (32)	21 (36)	19 (32)	NS
														
*bax*														
Friess *et al* (1998)	60	0.47 (0.23–0.97)	Yes	Santa Cruz (NS)	50 (83)	NS	32 (53)	63	38 (63)	NS	NS	NS	NS	NS
Evans *et al* (2001)	23	0.80 (0.28–2.29)	No	Santa Cruz (1 : 1600)	6 (26)	>5%	15 (65)	59	38 (63)	NS	5 (22)	13 (54)	5 (22)	0 (0)
Nio *et al* (2001b)	65	0.49 (0.28–0.85)	Yes	DAKO A3533 (1 : 100)	42 (65)	>10%	31 (47)	66	54 (82)	NS	33 (50)	29 (44)	4 (6)	36 (55)
Magistrelli *et al* (2006)	67	0.56 (0.33–0.95)	Yes	Zymed c2D2 (1 : 80)	36 (54)	>10%	45 (67)	63	34 (51)	40 (62)	14 (21)	28 (42)	15 (22)	30 (45)
Dong *et al* (2005b)	59	0.93 (0.57–1.52)	No	DAKO A3533 (1 : 100)	29 (49)	>10%	19 (32)	55	54 (82)	NS	19 (32)	21 (36)	19 (32)	NS
														
*p16*														
Naka *et al* (1998)	32	0.45 (0.21–0.96)	Yes	Santa Cruz C20 (1 : 500)	19 (59)	NS	20 (63)	65	23 (72)	13 (41)	NS	NS	NS	NS
Kawesha *et al* (2000)	157	0.82 (0.50–1.33)	No	Santa Cruz (1 : 100)	21 (13)	>5%	100 (64)	60	71 (46)	NS	21 (13)	77 (49)	59 (38)	13 (8)
Gerdes *et al* (2002)	40	0.51 (0.25–1.04)	No	Pharmingen G175–405 (1 : 50)	13 (33)	>5%	22 (55)	NS	16 (40)	NS	NS	NS	NS	0 (0)

Abbreviations: CI=confidence interval; HR=hazard ratio; IHC=immunohistochemical; NC=non-commercial; NS=not specified; VEGF=vascular endothelial growth factor.

% in parentheses unless otherwise stated. IHC and/or clinico-pathological data were incompletely reported in some studies. Well/Mod/Poor refers to tumour differentiation.

**Table 3:** Methodological and clinico-pathological data for eligible prognostic studies evaluating p53, smad4 and EGFR

**Reference**	***n***	**HR (95% CI)**	**Significant**	**1° Ab (+ dilution)**	**IHC +ve**	**IHC cutoff (%)**	**Male**	**Age**	**N1**	**T3/T4**	**Well**	**Mod.**	**Poor**	**Adjuvant therapy**
*p53*														
DiGiuseppe *et al* (1994)	48	1.77 (0.95–3.29)	No	Novocastra CM-1 (1 : 1000)	26 (54)	NS	25 (52)	61	NS	NS	NS	NS	NS	NS
Yokoyama *et al* (1994)	57	1.75 (1.01–3.02)	Yes	Novocastra DO7 (1 : 100)	33 (58)	NS	NS	64	25 (45)	27 (47)	37 (65)	20 (35)	NS	
Lee *et al* (1995)	26	1.17 (0.43–3.16)	No	Biogenex CM1 (NS)	7 (27)	NS	14 (54)	NS	NS	NS	2 (8)	20 (77)	4 (15)	NS
Linder *et al* (1997)	48	2.71 (1.41–5.20)	Yes	DAKO DO7 (1 : 50)	22 (46)	>1	36 (68)	66	18 (38)	26 (49)	5 (9)	18 (34)	30 (57)	NS
Virkajärvi *et al* (1997)	36	0.99 (0.46–2.11)	No	Novocastra CM-1 (1 : 1000)	15 (42)	>1	16 (44)	64	NS	NS	NS	NS	NS	NS
Naka *et al* (1998)	32	0.84 (0.34–2.10)	No	Novocastra BP53-12 (1 : 50)	19 (59)	NS	20 (63)	65	23 (72)	13 (41)	NS	NS	NS	NS
Bold *et al* (1999)	70	0.62 (0.34–1.12)	No	Oncogene DO1 (NS)	33 (47)	>25	36 (51)	64	38 (54)	NS	15 (22)	37 (56)	15 (22)	19 (27)
Gansauge *et al* (1999)	26	2.97 (1.30–6.79)	Yes	Oncogene DO1 (1 : 500)	11 (42)	NS	12 (50)	59	22 (85)	NS	NS	NS	NS	26 (100)
Ahrendt *et al* (2000)	43	0.77 (0.39–1.52)	No	DAKO DO7 (1 : 2000)	26 (60)	>33	24 (55)	63	23 (53)	22 (51)	11 (26)	23 (55)	8 (19)	29 (66)
Bergan *et al* (2000)	60	2.16 (1.14–4.08)	Yes	Novocastra DO7 (1 : 100)	15 (25)	>5	41 (50)	62	18 (30)	21 (35)	25 (42)	23 (38)	12 (20)	0 (0)
Kawesha *et al* (2000)	157	0.90 (0.64–1.26)	No	DAKO DO7 (1 : 300)	64 (41)	>5	100 (64)	60	71 (46)	NS	21 (13)	77 (49)	59 (38)	13 (8)
Gerdes *et al* (2002)	40	1.16 (0.61–2.21)	No	DAKO DO7 (1 : 400)	13 (33)	>10	22 (55)	NS	16 (40)	NS	NS	NS	NS	0 (0)
Sarela *et al* (2002)	52	0.53 (0.11–2.53)	No	DAKO DO7 (1 : 100)	28 (54)	>10	27 (52)	64	40 (78)	49 (94)	11 (22)	24 (47)	16 (31)	NS
Yamasawa *et al* (2002)	72	1.01 (0.62–1.66)	No	Oncogene DO1 (2 *μ*g/ml)	34 (47)	>20	34 (47)	65	21 (29)	42 (58)	35 (49)	32 (44)	5 (7)	41 (57)
Dong *et al* (2005a)	59	1.02 (0.60–1.73)	No	DAKO DO7 (1 : 20)	40 (68)	>10	38 (64)	NS	47 (80)	NS	19 (32)	21 (36)	19 (32)	NS
Magistrelli *et al* (2006)	67	0.68 (0.40–1.16)	No	DAKO DO7 (1 : 50)	32 (48)	>5	45 (67)	63	34 (51)	40 (62)	14 (21)	28 (42)	15 (22)	30 (45)
Jinfeng *et al* (2007)	32	3.18 (1.30–7.77)	Yes	DAKO DO7 (1 : 50)	13 (41)	>10	19 (59)	63	18 (56)	23 (72)	11 (34)	18 (56)	3 (10)	NS
														
*smad4*														
Tascilar *et al* (2001)	249	0.74 (0.56–0.98)	Yes	Santa Cruz B8 (1 : 100)	111 (46)	NS	139 (56)	65	NS	NS	NS	NS	NS	NS
Biankin *et al* (2002)	45	2.38 (1.08–5.24)	Yes	Santa Cruz B8 (NS)	10 (22)	>5	27 (60)	61	21 (47)	NS	5 (11)	28 (62)	12 (27)	8 (16)
Hua *et al* (2003)	34	0.94 (0.43–2.08)	No	Santa Cruz B8 (1 : 100)	26 (76)	NS	22 (65)	55	14 (41)	NS	27 (79)	7 (21)	NS	
Toga *et al* (2004)	88	0.51 (0.27–0.97)	Yes	Santa Cruz B8 (1 : 100)	13 (15)	>10	43 (49)	66	78 (89)	33 (37)	37 (42)	45 (51)	6 (7)	58 (66)
Khorana *et al* (2005)	124	0.88 (0.59–1.31)	No	Santa Cruz (1 : 400)	59 (48)	>5	69 (56)	67	56 (45)	69 (58)	23 (19)	52 (43)	45 (38)	88 (79)
														
*EGFR*														
Dong *et al* (1998a)	57	1.09 (0.51–2.32)	No	Oncogene 985/996 (1 : 20)	39 (68)	NS	20 (35)	55	46 (81)	NS	18 (32)	22 (39)	17 (30)	7 (12)
Ueda *et al* (2004)	76	2.25 (1.14–4.45)	Yes	Zymed 31G7 (1 : 200)	47 (62)	>10	57 (75)	63	59 (78)	NS	11 (14)	32 (42)	33 (43)	NS
Bloomston *et al* (2006)	71	0.79 (0.47–1.34)	No	Dakocytomation 218C9 (NS)	49 (69)	>1	40 (56)	65	41 (58)	57 (81)	6 (9)	45 (63)	20 (28)	NS
Smeenk *et al* (2007)	46	1.93 (0.92–4.07)	No	DAKO H11 (NS)	11 (24)	>1	37 (66)	63	29 (52)	34 (61)	6 (11)	43 (77)	7 (12)	19 (34)

Abbreviations: CI=confidence interval; EGFR=epidermal growth factor receptor; HR=hazard ratio; IHC=immunohistochemical; NC=non-commercial; NS=not specified.

% in parentheses unless otherwise stated. IHC and/or clinico-pathological data were incompletely reported in some studies. Well/Mod./Poor refers to tumour differentiation.
